# Evaluation of a Package of Behaviour Change Interventions (Baduta Program) to Improve Maternal and Child Nutrition in East Java, Indonesia: Protocol for an Impact Study

**DOI:** 10.2196/18521

**Published:** 2020-09-08

**Authors:** Michael John Dibley, Ashraful Alam, Umi Fahmida, Iwan Ariawan, Christiana Rialine Titaley, Min Kyaw Htet, Rita Damayanti, Mu Li, Aang Sutrisna, Elaine Ferguson

**Affiliations:** 1 Sydney School of Public Health The University of Sydney Australia; 2 SEAMEO RECFON-Pusat Kajian Gizi Regional Universitas Indonesia Jakarta Indonesia; 3 Center for Health Research Faculty of Public Health Universitas Indonesia Jakarta Indonesia; 4 Faculty of Medicine Pattimura University Ambon City Indonesia; 5 Indonesia Office Global Alliance for Improved Nutrition Jakarta Indonesia; 6 Department of Population Health London School of Hygiene & Tropical Medicine (LSHTM) London United Kingdom

**Keywords:** infant, feeding behavior, diet, food, and nutrition, growth disorders, undernutrition, nutrition during pregnancy, water treatment

## Abstract

**Background:**

Over the past decade, the prevalence of stunting has been close to 37% in children aged <5 years in Indonesia. The Baduta program, a multicomponent package of interventions developed by the Global Alliance for Improved Nutrition, aims to improve maternal and infant nutrition in Indonesia.

**Objective:**

This study aims to assess the impact of the Baduta program, a package of health system strengthening and behavior change interventions, compared with the standard village health services on maternal and child nutrition.

**Methods:**

The impact evaluation uses a cluster randomized controlled trial design with 2 outcome assessments. The first uses cross-sectional surveys of mothers of children aged 0-23 months and pregnant women before and after the interventions. The second is a cohort study of pregnant women followed until their child is 18 months from a subset of clusters. We will also conduct a process evaluation guided by the program impact pathway to assess coverage, fidelity, and acceptance. The study will be conducted in the Malang and Sidoarjo districts of East Java, Indonesia. The unit of randomization is the subdistricts. As random allocation of interventions to only 6 subdistricts is feasible, we will use constrained randomization to ensure balance of baseline covariates. The first intervention will be health system strengthening, including the Baby-Friendly Hospital Initiative, and training on counseling for appropriate infant and young child feeding (IYCF). The second intervention will be nutrition behavior change that includes *Emo-Demos*; a national television (TV) advertising campaign; local screening TV spots; a free, text message service; and promotion of low-cost water filters and hygiene practices. The primary study outcome is child stunting (low length-for-age), and secondary outcomes include length-for-age Z scores, wasting (low weight-for-length), anemia, child morbidity, IYCF indicators, and maternal and child nutrient intakes. The sample size for each cross-sectional survey is 1400 mothers and their children aged <2 years and 200 pregnant women in each treatment group. The cohort evaluation requires a sample size of 340 mother-infant pairs in each treatment group. We will seek *Gatekeeper* consent and written informed consent from the participants. The intention-to-treat principle will guide our data analysis, and we will apply Consolidated Standards of Reporting Trials guidelines for clustered randomized trials in the analysis.

**Results:**

In February 2015, we conducted a baseline cross-sectional survey on 2435 women with children aged <2 years and 409 pregnant women. In February 2017, we conducted an end-line survey on 2740 mothers with children aged <2 years and 642 pregnant women. The cohort evaluation began in February 2015, with 729 pregnant women, and was completed in December 2016.

**Conclusions:**

The results of the program evaluation will help guide policies to support effective packages of behavior change interventions to prevent child stunting in Indonesia.

**International Registered Report Identifier (IRRID):**

RR1-10.2196/18521

## Introduction

### Background

Over the past decade in Indonesia, there has been little change in child stunting, with the national prevalence of stunting estimated as 37% of children aged under 5 years [1,2]. This high level of growth failure has profound adverse consequences for the physical and economic development of affected children, their communities, and the country overall [3-7]. Growth faltering among Indonesian children starts in utero and is associated with low maternal height and poor nutritional status [8-15]. Among children in Indonesia, as elsewhere, 2 major determinants—undernutrition and infections—broadly account for postnatal deterioration in growth [10,15-20]. In studies examining risk factors for child stunting in Indonesia, lower maternal education and household wealth, including lower expenditure on foods from animal sources, are associated with a higher prevalence of child stunting [15,18,19,21].

Presidential Decree Number 42/2013 on The National Movement for the Acceleration of Nutrition Improvement prioritized the improvement of nutritional status in the first 1000 days of life, with the stunting of children aged <5 years as a key indicator. The decree addresses 4 main strategies. The first is to make nutritional improvements as the mainstream of the development plan. The second is building capacity and increasing human resource competence to respond to nutritional needs in all sectors. The third is to promote effective evidence-based interventions to improve nutrition. The fourth is increasing community participation in nutrition programs. The Government of Indonesia prioritized stunting reduction as part of the Ministry of Health Strategic Plan 2015-2019 and aimed to reduce the prevalence of stunting among children aged <2 years from 33% in 2013 to 28% in 2019 [22].

In Indonesia, the public health system is decentralized and is the responsibility of the district and municipal governments. They manage health services through the District Health Office and deliver primary health care services through community health centers (*Puskesmas*). Civil society also actively participates in the health sector through a community initiative called the integrated village health post (*Posyandu).* It consists of community volunteers (health cadres), trained by village midwives, or other community health center staff. The health cadres assist with preventive health services, including nutrition education to pregnant women and caregivers of children aged 0-59 months.

In 2014, the Ministry of Health requested the Global Alliance for Improved Nutrition (GAIN) to support the Malang and Sidoarjo districts in East Java to reduce child stunting. Subsequently, GAIN initiated the *Baduta* (means children aged <2 years in Indonesian) program with funding from the Ministry of Foreign Affairs of the Netherlands and in collaboration with Save the Children, Paramitra Foundation, and PT Holland for water (Nazava). The experience and recommendations generated through the Baduta program will inform on efforts to improve nutrition and prevent stunting nationally in Indonesia. GAIN invited the University of Sydney, in collaboration with the Centre for Health Research, Universitas Indonesia, and the London School Hygiene and Tropical Medicine to conduct a comprehensive theory-driven evaluation designed to learn about the factors affecting program delivery and the achievement of the impact of the interventions [23,24]. This paper describes the study protocol for the impact and process evaluations of the Baduta program.

### Objectives

The Baduta study aims to assess the impact of a package of health system strengthening and behavior change interventions on maternal and child nutritional status compared with the standard, integrated village health post services. The primary outcome in the cross-sectional evaluation will be the prevalence of low length-for-age (<-2 Z scores) in children aged <2 years in each treatment group, whereas in the cohort evaluation, it will be the mean length-for-age Z scores in each treatment group. The secondary outcomes will include mean length-for-age Z scores, the prevalence of low weight-for-length (<-2 Z scores), child anemia (hemoglobin [Hb] <11 g/dL), indicators of infant and young child feeding (IYCF) practices, maternal and infant dietary intake, and use of iron and folic acid supplements by pregnant women.

### Hypotheses

#### Primary Hypothesis

A package of behavior change interventions and health system strengthening activities delivered to a rural population in East Java, Indonesia, over 18 months will improve women’s diet and iron supplementation in pregnancy, breastfeeding, and complementary feeding practices; promote hygienic food preparation; and promote use of clean drinking water. The interventions will reduce the prevalence of stunting (length-for-age Z score <-2 Z) in children aged 0 to 23 months by 6% (35% in the control group to 28.9% in the intervention group) as assessed in cross-sectional surveys, compared with areas with only the routine integrated village health post services.

#### Secondary Hypotheses

The interventions will (1) increase mean length-for-age Z scores, mean weight-for-age Z scores, and mean weight-for-length Z scores at 18 months in the cohort of children followed up longitudinally; (2) decrease the prevalence of low weight-for-age and low weight-for-length in baseline and end-line cross-sectional surveys; (3) decrease the number of children ill with diarrhea, acute respiratory illness, or fever in both cross-sectional and cohort assessments; (4) decrease the prevalence of anemia in pregnant women and children aged <2 years; (5) increase women’s knowledge about appropriate breastfeeding practices; (6) increase median duration of exclusive breastfeeding and the percentage of women exclusively breastfeeding at 6 months and age-appropriate breastfeeding in children aged 0 to 23 months; (7) increase women’s knowledge about appropriate complementary feeding including snacks; (8) decrease the percentage of children aged <2 years receiving snacks; (9) increase the percentage of children consuming ≥4 food groups at ages 6-8, 9-11, and 12-23 months in the end-line cross-sectional assessment and ages 7, 10 and, 17 months in children followed up in the cohort assessment; and (10) increase nutrient intakes, nutrient density, and dietary adequacy for children aged 6-23 months.

## Methods

### Study Design

**Figure 1:**
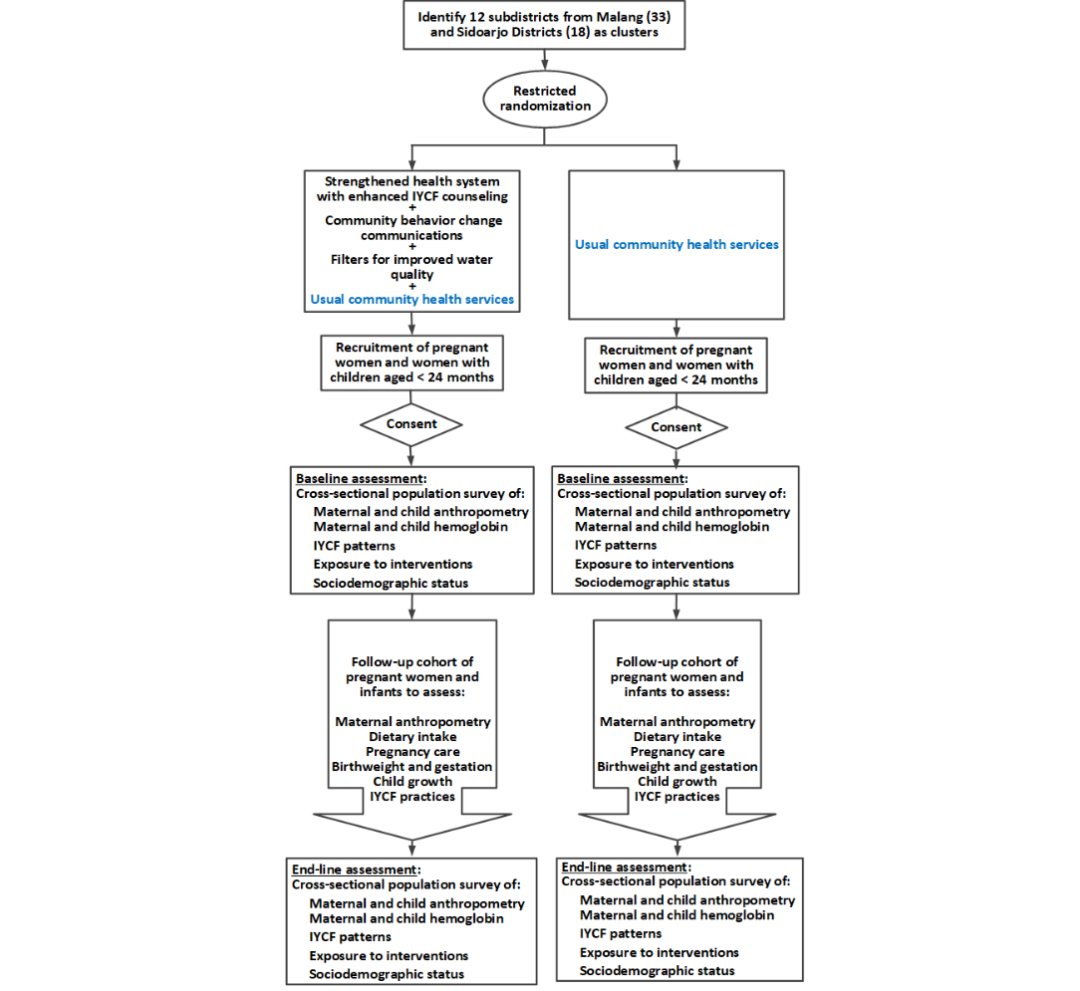
Diagram of the study design for the impact evaluation. IYCF: infant and young child feeding.

The study is a probability impact evaluation [25] using a cluster randomized controlled trial with 2 parallel treatment arms. The design uses a superiority hypothesis, having one-to-one allocation of the treatments, 2 types of outcome assessments, and a process evaluation (Figure 1).

#### Cross-Sectional Survey Assessment

The first assessment approach will use repeated cross-sectional surveys of mothers of children aged 0-23 months and pregnant women at baseline before interventions commence and 2 years later at the end line, which is the best approach to assess the impact of the intervention on the stunting prevalence in the study population (population-level impact) [26]. In the cross-sectional surveys, we will collect information on socioeconomic and demographic characteristics, infant feeding practices including intentions of the mother to breastfeed and mother’s self-efficacy with breastfeeding, child morbidity reported by mother/caregiver, contact with the health system and exposure to the interventions, maternal and child anthropometry, and maternal and child hemoglobin. We will assess the impact by comparing the change in the prevalence of key indicators between the treatment groups in children aged <2 years and pregnant women.

#### Cohort Study Assessment

The second will be a cohort study that will recruit pregnant women from a subset of study clusters and follow them from late in the third trimester of pregnancy until their child is 18 months old to examine the impact of the interventions on child growth at the individual level. This cohort study will allow us to examine subgroups for any modifying effects on the primary outcome, for example, the modifying effects of different levels of maternal education and household wealth on the impact of the intervention on the study outcomes. We will exclude women who are severely anemic (Hb<7 g/dL) or have chronic diseases such as tuberculosis, women who intend to migrate outside the subdistrict, infants born with a visible congenital defect, and multiple births (twins) from the cohort study.

### Program Impact Pathways

**Figure 2:**
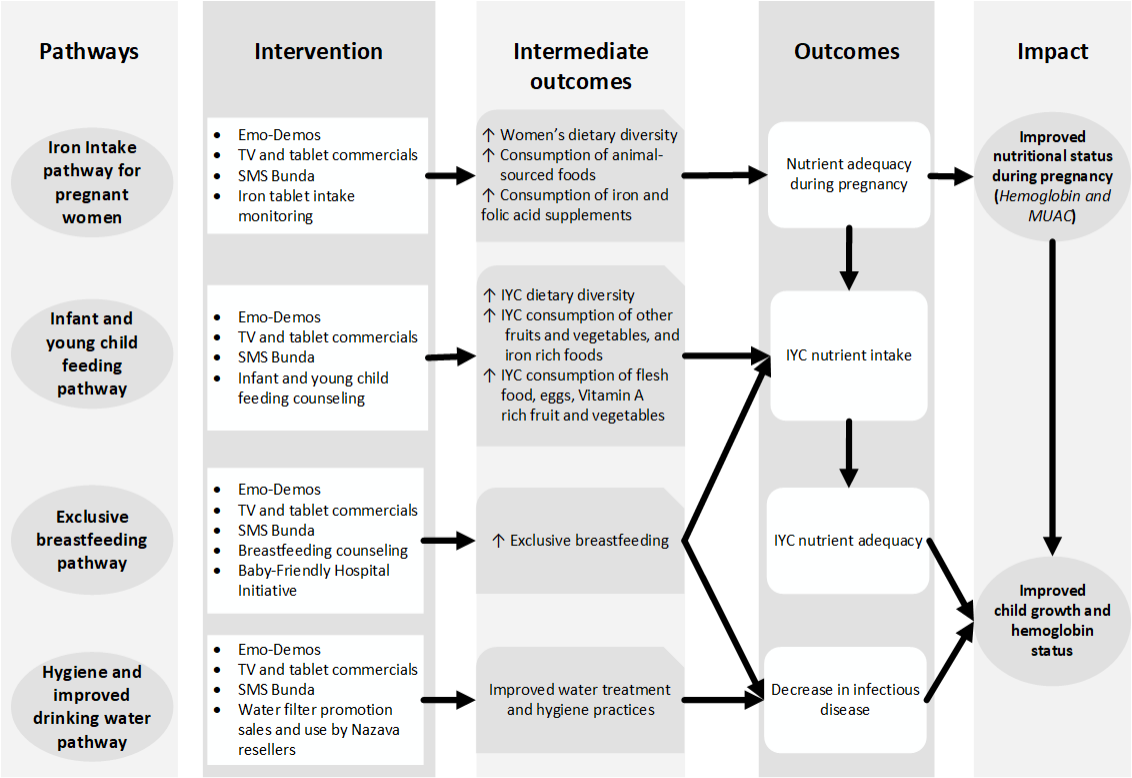
Impact pathways of the Baduta program interventions. IYC: infant and young child; TV: television.

The Baduta interventions should operate through 4 intervention pathways, as shown in the program impact pathway illustrated in Figure 2. The first pathway is to improve nutritional status during pregnancy by improving the nutrient adequacy of diets through increased consumption of foods from animal sources and iron and folic acid supplements. The second is to improve the nutrient adequacy of infant and young child diets through improved dietary diversity. The third is to reduce infectious diseases and improve nutrient intake through adherence to exclusive breastfeeding in the first 6 months of life. The fourth is to reduce infectious diseases through improved water and sanitation practices.

### Process Evaluation

During the delivery of the interventions, we will also conduct a process evaluation to assess the intervention input, output, and outcomes related to the program impact pathway and responses of the target populations to the intervention and contextual factors affecting the intervention. We will integrate and align the process evaluation with process monitoring of the implementers of the interventions.

### Study Setting

We will perform the study in the Malang and Sidoarjo districts of East Java, Indonesia (see Multimedia Appendix 1—a map of East Java with the study districts marked), which had an estimated population of almost 2 million and 2.5 million in 2014, respectively. There are 350 villages in Sidoarjo and 390 villages in Malang. The population density of Sidoarjo (2891/km^2^) is almost 4 times the population density of Malang (780/km^2^) [27]. Sidoarjo is a peri-urban district, with 13% of households from urban areas, and most adults are employed as factory workers (36%) or providing public and private services (41%: trader, hotels, restaurant, and public institutions) [28]. Malang is a rural district, with 97% of households from rural areas, and most adults are employed as farmers (33%) or providing public and private services (34%: traders, hotels, restaurants, and public institutions) [29]. On the basis of the annual nutrition surveillance data from the Ministry of Health, there are 24% of children aged <5 years who are stunted in Sidoarjo and 31% in Malang. Approximately two-thirds of the children aged <5 years in Malang and a half in Sidoarjo attend integrated village health posts for weight monitoring [28,29].

There are 51 subdistricts available for the study (33 in Malang and 18 in Sidoarjo), with an average population of 86,000. On average, there are 1.3 community health centers in each subdistrict, and each covers a population of approximately 68,000 people. Within the catchment area of each community health center, there are, on average, 2.8 subcenters (*Pustu*) that cover 5 villages or about a population of 30,000. Primary health care outreach to the community uses monthly integrated village health posts organized by local female health volunteers who coordinate with the community health center staff and village midwives. Village midwives are trained midwives who reside in 1 village within the community health center catchment area and are part of the service network. The village midwives’ program is part of the government’s efforts to accelerate the improvement of community health, particularly concerning maternal and child health. The roles and responsibilities of village midwives include maternal and child health, family planning services, health promotion, and preventive services as well as early detection and initial treatment for maternal and child health conditions, including undernutrition.

### Cluster Selection and Design of Randomization

The unit of randomization for the study will be subdistricts, selected by the authors to prevent contamination as they will deliver the health system strengthening interventions through the subdistrict community health centers. It will only be feasible to randomize a limited number of subdistricts to the new interventions versus the standard programs. As we will be limited to 12 subdistrict clusters (6 intervention and 6 usual programs), it will not be possible to use simple randomization to allocate them randomly to the intervention or control groups.

We will use constrained randomization [30,31] to ensure a balanced distribution of covariates in the study treatment groups because of the limited number of subdistrict clusters in which it is feasible to conduct the study. There are 33 subdistricts in Malang and 18 subdistricts in Sidoarjo. We will exclude subdistricts with similar ongoing projects and small subdistricts (population <70,000), leaving 24 subdistricts eligible for the study.

We will conduct hierarchical cluster analysis on the 24 eligible subdistricts using data for each district from the 2011 Village Potential Statistics (Pendataan Potensi Desa/Kelurahan) data set and local health surveillance data. We will use indicators of household economic status, access to health care, and prevalence of undernutrition in children to identify a group of similar subdistricts. We will select a group of 12 of the most similar subdistricts and construct a list of the 924 possible combinations of 6 intervention subdistricts and 6 comparison subdistricts. We will perform the analyses for constrained randomization with SAS 9.2 (SAS Institute Inc) using an algorithm developed by Chaudhary and Moulton [32].

We will select combinations of intervention and comparison subdistricts with balanced covariates, as measured by a difference in (1) the average distance of the neighborhood to the closest community health care center with a cutoff value for balance <0.5 km, (2) the coverage of children weighted at village health posts with a cutoff value for balance <5%, (3) the prevalence of underweight children (weight-for-age less than –2 Z score from growth monitoring charts) with a cut off value for balance <2.5%, (4) the ratio of village health posts to the total population with a cutoff value for balance <1.6/10,000 population, and (5) the percentage of low-income families who have a local government Certificate of Disadvantage (Surat Miskin/Surat Keterangan Tidak Mampu) with a cutoff value for balance <5%. Using simple random sampling, we will select one of the balanced combinations of subdistricts for allocation of the study interventions.

### Formative Research and Pilot Studies

Before designing the Baduta program, GAIN conducted formative research to identify delivery platforms, create communication strategies and frame messages, and develop communication materials [33]. The GAIN staff and a research team from the London School of Hygiene and Tropical Medicine, London, United Kingdom, and the Regional Centre for Food and Nutrition, Southeast Asian Ministers of Education Organization (SEAMEO-RECFON), Jakarta, Indonesia, conducted formative research and a pilot study of the Healthy Gossip Movement (Gerakan Rumpi Sehat) intervention [33] to determine whether a communication strategy that focused on strong behavioral handles rather than educational messages would be effective in changing nutritional behavior. The Baduta project team developed 12 participative and demonstrative activities for delivery in community settings called *Emo-Demos*. The *Evo-Eco* theory [34] underpins these activities, which are grounded in the psychological and environmental determinants of behavior. This approach suited a community setting where nutrition knowledge was high, but the adoption of healthy nutritional behaviors was poor [35]. The pilot study found a greater improvement in dietary diversity when messaging based on emotional drivers of behavior change was channeled through both mass media TV adverts and *Emo-Demo* community activities [33]. We used these 2 interventions in the package of behavior change interventions for the Baduta program.

### Description of the Baduta Program Interventions

The interventions will primarily aim to strengthen the existing activities delivered through the public health system to promote recommended nutrition practices for pregnancy, breastfeeding, and complementary feeding. We will implement the following activities in the intervention subdistrict clusters during the study.

#### Health System Strengthening Interventions

In the first health system strengthening intervention, Save the Children will launch the implementation of the Baby-Friendly Hospital Initiative (BFHI) [36] in the public sector district and municipal hospitals and birthing centers at the community health centers in the intervention subdistricts. The World Health Organization (WHO) and the United Nations Children’s Emergency Fund (UNICEF) jointly conceived the BFHI in 1991 as a strategy to improve breastfeeding rates worldwide. A hospital or birth center can receive Baby-Friendly designation if they show compliance with the Ten Steps to Successful Breastfeeding [36].

In the second health system strengthening intervention, Save the Children will train village midwives, health workers, and integrated village health post cadres on counseling for appropriate IYCF using the Indonesian Ministry of Health adaptation of the WHO/UNICEF Community Infant and Young Child Feeding Counseling Package [37].

#### Behavior Change Interventions

We will employ 4 different behavior change interventions to address program outcomes, as shown in the program impact pathway (Figure 1). We have developed behavior change communications using the Behavior Centered Design theory and employed emotional drivers of behavior change rather than education-focused messaging [33].

The first will be 12 participative and demonstrative behavior change activities called *Emo-Demos*, developed by the London School of Hygiene and Tropical Medicine, London, United Kingdom, and GAIN [33], which are based on the psychological and environmental determinants of behavior. The Emo-Demos include topics on nutrition during pregnancy (3 activities), breastfeeding (3 activities), care during pregnancy (1 activity), complementary feeding issues (4 activities), and handwashing (1 activity; see Multimedia Appendices 2 and 3 for details of Emo-Demos). The Paramitra Foundation will recruit and train village facilitators to train village midwives and health cadres to deliver Emo-Demos during monthly pregnant women classes and child growth monitoring at the integrated village health post.

The second will be a national TV campaign that will have 4 high-quality spots with messages on nutrition during pregnancy (1 spot), breastfeeding (1 spot), and complementary feeding issues (2 spots; see Multimedia Appendices 4 and 5 for a description of these TV spot messages). The TV spots will air on 5 national TV channels, and it will not be possible to contain them to only the intervention subdistricts. Village facilitators will also screen the TV spots using tablets during integrated village health post meetings and with people passing by during *street visits*.

The third will be the established SMS Bunda intervention, a free, text message service for pregnant women and postnatal mothers that aims to reduce maternal and infant mortality [38]. Midwives encourage women to access the service by registering their mobile number and the expected date of delivery at any time during pregnancy. Initially, in pregnancy, the women receive regular short messages about antenatal care. In the last month of pregnancy, the text message contains information related to the first days after childbirth for mother and baby, including information about early initiation of breastfeeding. In the first month of birth, the messages provide information about the health of mothers and newborns, including exclusive breastfeeding.

In the fourth intervention, GAIN will partner with a company, Nazava, to expand sales of low-cost water filters and promote appropriate water treatment and hygiene practices. Water filters are a low-cost and effective method to purify water that avoids costly fuels (wood or electricity) to boil water. Nazava’s rural sales network in the Malang and Sidoarjo districts as well as trained resellers will market the filters and provide education sessions on water treatment and handwashing.

### Description of Standard Primary Health Care Services

During the study, both the intervention and the comparison clusters will receive the standard primary health care services. Monthly integrated village health posts are the delivery platforms for the usual community health services that include child growth monitoring, food supplements, child immunizations, antenatal care, pregnancy classes, and family planning services. The local community, particularly the village health cadres, organize the integrated village health post services under the supervision of the community health center staff. The community health center staff, village midwives, and village health cadres deliver the services.

### Evaluation Outcomes

#### Primary Outcomes

The primary outcome for the cross-sectional evaluation is the difference in the percentage of stunted (length-for-age<–2 Z scores) children aged 0-23 months between the intervention and the comparison groups at the baseline and end-line surveys. For the cohort evaluation, the primary outcome is the difference in the mean length-for-age Z scores between the intervention and the comparison groups every 3 months from birth to 18 months, as measured in the follow-up assessments.

#### Secondary Outcomes

The secondary outcomes for the cross-sectional evaluation include differences between the 2 treatment groups at the baseline and end-line surveys in the following: (1) the percentage of wasted (weight-for-length<–2 Z scores) children aged 0-23 months and their mean weight-for-length Z scores; (2) the percentage of children aged 0-23 months with low hemoglobin (Hb <11 g/dL) and their mean hemoglobin; (3) the number of events and the mean number of days the children are ill >2 weeks before interview with diarrhea, acute respiratory infections, and fever; (4) the percentage of children aged 0-23 months who are ever breastfed, are breastfed with an hour of birth, are given prelacteal feeds, are exclusively breastfed in the first 6 months of life, continue breastfeeding at 1 and 2 years, and are age appropriately breastfed; (5) the percentage of children aged 6-23 months with minimum dietary diversity, minimum meal frequency, minimum acceptable diet, and frequency of consumption of 7 food groups and iron-rich foods; (6) the percentage of children aged 6-23 months consuming snacks the day before the interview; (7) the mean intake of food energy, protein, carbohydrate, fat, and selected micronutrients from complementary foods and the micronutrient density of the complementary feeding diets compared with desired levels at 6-8, 9-11, and 12-23 months of age; (8) the percentage of children aged 12-23 months at risk of inadequate nutrient intake; (9) the percentage of mothers with appropriate knowledge about IYCF; and (10) the percentage of mothers with high self-efficacy for breastfeeding.

The secondary outcomes for the cohort evaluation include differences between the 2 treatment groups in the following: (1) the percentage of wasted (weight-for-length<–2 Z scores) children and their mean weight-for-length Z scores every 3 months from 3 to 18 months; (2) the percentage of children with low hemoglobin (Hb <11 g/dL) and their mean hemoglobin at 18 months of age; (3) the number of events and the mean number of days the children are ill >2 weeks before interview with diarrhea, acute respiratory infections, and fever from birth to 18 months; (4) the percentage of children who are ever breastfed, are breastfed within 1 hour of birth, are given prelacteal feeds, and are exclusively breastfed each month from birth to 6 months; (5) the median maternal breastfeeding self-efficacy score and the percentage of mothers with high self-efficacy for breastfeeding at 7 days and 3 and 6 months postpartum; (6) the percentage of children with minimum dietary diversity, minimum meal frequency, and minimum acceptable diet at 7, 10, and 17 months of age; (7) the percentage of children consuming snacks within 1 hour of meals and the contribution of snacks to energy intake at 6-8, 9-11, and 16-18 months of age; (8) the mean intake of food energy, protein, carbohydrate, fat, and selected micronutrients from complementary foods and the micronutrient density of the complementary feeding diets compared with desired levels at 6-8, 9-11, and 12-23 months of age; (9) the percentage of women receiving and consuming iron and folic acid supplements during pregnancy; and (10) the percentage of women pregnant in their third trimester meeting minimum dietary diversity and at risk of inadequate nutrient intakes.

### Outcome Measurements

In the cross-sectional assessment at baseline and end-line surveys, we will measure the following factors or indicators: social, economic, and demographic characteristics; infant feeding practices; child morbidity; contact with the health system; and exposure to the interventions. We will also measure maternal and child anthropometry and hemoglobin status.

In the cohort assessment, we will conduct repeated data collection on social, economic, and demographic characteristics; infant feeding practices; maternal and child dietary intake; child morbidity; contact with the health system; and exposure to the interventions. We will also conduct repeated measurements of maternal and child anthropometry and child hemoglobin status at 18 months.

#### Anthropometry

Trained field workers will collect measurements of weight, recumbent length in infants, height in mothers, and mid-upper arm circumference in pregnant women using standard methods and equipment [39,40]. We will standardize the measurements of the anthropometry field workers using established methods before and during data collection [39]. In both the cross-sectional and cohort evaluations, 2 field workers will collect duplicate measurements of the weight and length of children aged <2 years. The field workers will take a third measurement if the difference between the first 2 measurements exceeds a predetermined allowable limit (see details below).

We will calculate the Z scores for infant length-for-age and weight-for-length using the 2006 WHO Child Growth Standard [41], and we will define stunting as a low length-for-age or <−2.00 SD from the reference mean and wasting as low weight-for-length or <−2.00 SD. We will collect mid-upper arm circumference from pregnant mothers and define it as low if <23 cm [42].

The field workers will collect all anthropometry data using in-field digital data capture on tablets with routines that immediately calculate Z scores and provide them with a warning about extreme values and the need for remeasurement to check for potential errors (ie, if first and second measurement differed by >0.7 cm for length and by >0.1 kg for weight). We will refer to children with very low weight-for-height (Z score<−3) to their local community health center for assessment and treatment of severe acute malnutrition.

#### Birth Weight and Duration of Gestation

In the cross-sectional assessment, we will ask mothers of children aged <2 years to recall the birth weight and ask them to describe their infant’s birth size using the same question as in the Indonesian Demographic and Health Survey (DHS) [43]. There will be no estimate of the duration of gestation. In the cohort assessment, we will measure birth weight within 72 hours after birth. We will estimate the duration of gestation using the mother’s report of the first date of her last menstrual period.

#### Hemoglobin

In the cross-sectional evaluation, we will assess hemoglobin in all women and children. However, in the cohort evaluation we will assess hemoglobin from children at 18 months and mothers during pregnancy. We will collect capillary blood samples and measure hemoglobin using a portable hemoglobinometer (Hemocue). We will refer mothers with a hemoglobin level <12 g/dL and children with a hemoglobin level <11 g/dL to their local community health center for assessment and treatment.

#### Child Morbidity

**Figure 3:**
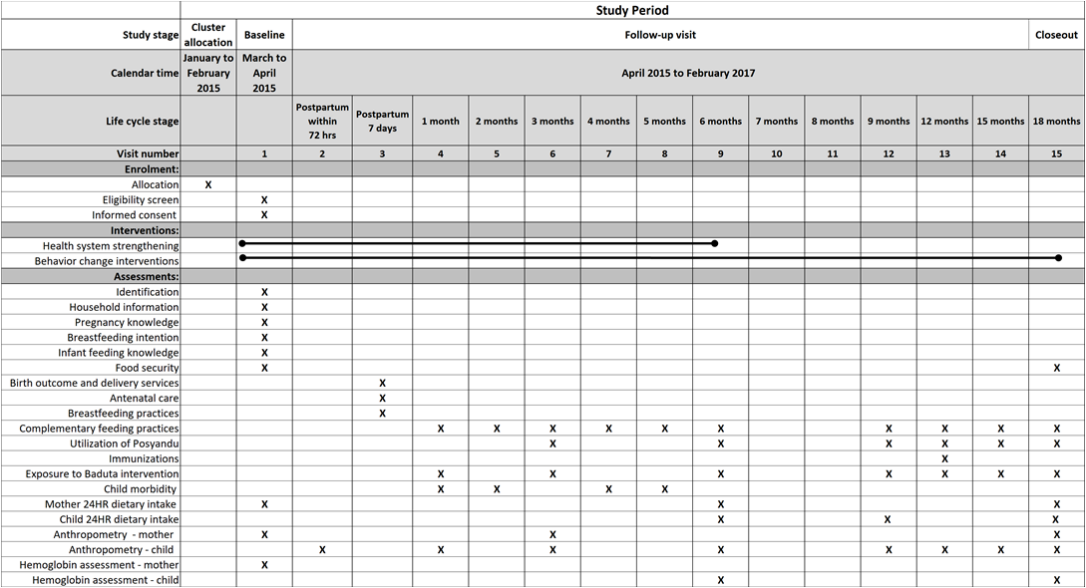
Schedule of enrollment, interventions, and assessments for cohort evaluation. X: indicates activity occurred at given visit or life cycle stage; lines mark the duration of the interventions; 24 HR: 24-hour dietary recall.

In the cross-sectional and cohort assessments at the scheduled visits (Figure 3), trained interviewers will record maternal recall of symptoms from the preceding 2 weeks of common childhood illnesses (diarrhea, cough, and fever) using the standard Indonesian DHS questions.

#### IYCF

In both the cross-sectional and cohort evaluations, we will collect information about IYCF using an interviewer-administered questionnaire on breastfeeding and general complementary feeding practices. In the cross-sectional evaluation, trained interviewers will ask questions about infant feeding practices based on questions from the Indonesian DHS that allow estimation of the standard WHO IYCF indicators [44]. These IYCF questions will include the timing of initiation of breastfeeding; current breastfeeding status; use of bottles for feeding; current use, timing, and introduction of other liquids and solid foods; meal frequency; and consumption of food groups in the previous 24 hours and week before the interview. We will also collect 24-hour dietary recalls (24-HR), which will be used to assess indicators of IYCF (see the Dietary Intake Data section).

In the cohort assessment, we will administer the questions about IYCF at the same time as we collect the anthropometric data, that is, soon after birth, every month in the first 6 months, then every 3 months until they are 18 months of age.

We will estimate indicators of IYCF according to the WHO indicators for assessing IYCF practices [44], including early initiation of breastfeeding, exclusive breastfeeding, predominant and continued breastfeeding, bottle-feeding practices, dietary diversity score, meal frequency, minimal acceptable diet, and timing of initiation of complementary feeding.

Information will be collected about mothers’ intentions to breastfeed their infants when they are pregnant. We will assess the mothers’ self-efficacy with breastfeeding using the breastfeeding self-efficacy short questionnaire developed by Dennis [45], which is a 14-item instrument aimed at measuring breastfeeding confidence, at birth and at 1, 3, and 6 months postpartum.

#### Dietary Intake Data

Quantitative dietary intake data will be collected using 4-pass 24-HR. In the cross-sectional evaluation, we will collect it at baseline and end line from pregnant women and infants aged 6-23 months, whereas in the cohort assessment, we will collect it from mothers during pregnancy (third trimester) and from infants at 7, 10, and 17 months of age. Paper-based data collection was used to collect dietary data in the cross-sectional surveys and for the pregnant women in the cohort study, whereas digital data capture was used to collect the 24-HR from children in the cohort study.

For each target group and data collection period, 24-HRs will be proportionately collected on all days of the week to account for any day of the week effects on dietary intakes. In subsamples of pregnant women and infants aged 12-23 months, we will collect repeated 24-HRs on nonconsecutive days (n=40 per target group per data collection period) to adjust nutrient intake distributions for intrasubject variability [46] when estimating the percentage at risk of inadequate nutrient intakes.

We will collect all 24-HRs in the participants’ homes by trained interviewers using standardized methods [46]. Briefly, in the *first pass*, each respondent will be asked to recall all foods and beverages consumed by herself (or her child) during the preceding day. In the *second pass*, for each food and beverage listed, we will record details about the time of consumption, the food and beverage (brand names, ingredients, and cooking methods), and any food items added (eg, sugar added to tea). In the *third pass*, we will ask the respondents to estimate the amount they (or their child) consumed, after probing for leftovers. Portion sizes will be quantified by weighing the indicated amounts of real foods, photographs, food models, or by the number of standard units consumed. We will develop a standard portion size manual to ensure that all interviewers use consistent methods. We will record recipes, including the amounts of raw food used in the recipe, the cooking method, and the total amounts cooked and consumed. In the *fourth pass*, the interviewers will review the dietary data for completeness, and they will ask whether the previous day’s diet represented a typical day and, if not, how it differed from usual, and for infants, whether they were breastfed on the previous day.

#### Dietary Data Processing

Dietary data will be collected using either a paper-based questionnaire or an in-field digital data capture on tablets for multiple-pass 24-HR that is under development. The digital data capture system saves data processing time and reduces errors by ensuring that probes and food portion size estimations are standardized. At the end of each data collection day, all dietary data will be checked for completeness by the team supervisor. Paper-based questionnaires will be entered into computer files with special-purpose data entry screens, and digitally captured questionnaires will be downloaded directly to a central server. The 24-HR records from both sources will be reviewed by trained nutritionists to monitor data quality and to select the appropriate food composition for foods not linked to an existing food composition item. We will monitor dietary data quality by applying a plausible energy range for children (by age group and breastfeeding status) to identify energy under- and over-reporting.

### Process Evaluation

Process evaluation of the Baduta study aims to assess program coverage, fidelity, and acceptance by the intended target population, guided by the program impact pathway, and to demonstrate plausibility and the attribution of impacts to the intervention. We will emphasize the 4 program impact pathways that include knowledge adoption of (1) exclusive breastfeeding, (2) IYCF practices, (3) iron intake pathway, and (4) knowledge adoption of household water treatment and safe storage and hand washing. In the process evaluation, we will collect data about the interventions attached to these 4 pathways, such as the BFHI, breastfeeding and complementary feeding counseling, emotional demonstration (Emo-Demo), and enhanced water treatment through the household-level promotion of Nazava water filters.

We will use mixed methods to collect process evaluation data. The qualitative methods will include semistructured interviews with women participating in the intervention, health workers (midwives, nurses, village midwives, cadres, nutritionists, and doctors), managers (the head of the community health center and the head of midwives), and intervention implementers (Project Manager, Monitoring Officer, and Field Officer of Save the Children; Director, Project Manager, Monitoring Officer, and Subdistrict Coordinator of Paramitra; and Project Manager, Safe Eater Consultant, and Save Water Entrepreneur of Nazava); a structured questionnaire survey of the water filter users; and observation of Emo-Demo sessions. Apart from these primary data, we will use secondary data from the project monitoring records maintained by Save the Children, Paramitra, and Nazava.

The field research team will consist of nutritionists, anthropologists, and psychologists who have previous experience in qualitative research and speak the local language. The investigators from the University of Indonesia, SEAMEO-RECFON, and the University of Sydney will train the field research team in qualitative data collection, data organization methods, and survey methods. They will be involved in finalizing the data collection tools that will be pretested before data collection. We will purposively select a sample of the clusters from the evaluation study to collect the process evaluation data.

### Sampling Design and Sample Size

#### Sample Size

We estimated the targeted sample size for the study using the following assumptions: (1) in the 51 subdistricts in the Malang and Sidoarjo districts, there is a total population of approximately 4.4 million, with an average of approximately 86,000 per subdistrict; (2) assuming a crude birth rate of 17 per 1000 population per annum, 5% refuse, and 10% data losses, each subdistrict cluster would yield an average of 620 live births over 6 months; (3) an expected prevalence of stunting for children aged 0-23 months of 35% in the control clusters (based on unpublished survey data from Brebes, West Java, and Indonesian DHS data); (4) 80% power and 5% 2-sided alpha, and 1:1 ratio of interventions (subdistricts with and without the Baduta program) allocated; (5) design effect of 1.5 (lower than reported for the 2011 Indonesia DHS survey because of larger sized clusters); and (6) expected percentage point difference in the prevalence of stunting (0-23 months) between the intervention groups of 6% (35.0% in the control to 28.9% in the intervention group). (This is a relative reduction of 17%, which is lower than the 29% reduction in stunting reported in a recent meta-analysis [47] of education interventions to improve complementary feeding.)

A standard formula using established methods [48] for comparison of proportions to calculate the number of subjects required per intervention arm to test H_0_: *π*_0_=*π*_1_ is as follows:

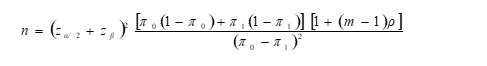



where *z_α_*_/2_ and *z_β_* are standard normal distribution values for upper tail probabilities of *α*/2 and *β,* respectively, *π*_0_ is the proportion in control, *π*_1_ is the expected proportion in intervention, *m* is the number of individuals per cluster, and *ρ* is the intracluster correlation coefficient.

Based on this calculation, the sample size required is 1392 mother-infant dyads from each group of 6 subdistrict clusters or approximately 232 mother-infant dyads per subdistrict cluster. We will round this up to 2800 mother-infant dyads per cross-sectional survey or 234 per subdistrict cluster. This sample size will provide 80% power assuming a Z score SD of 0.975 and 5% level of significance to detect a 0.15 Z score difference in height-for-age Z scores [49] between the intervention and the control groups in the end-line survey. Therefore, for the cross-sectional evaluation, we will select 2800 mothers of children aged 0-23 months and an additional 400 pregnant women from 12 subdistricts within the Sidoarjo and Malang districts.

For the cohort evaluation, to detect a difference in mean length-for-age Z score between the experimental and control groups at an end line of 0.35 Z score [50], assuming 80% power and 5% level of significance, with a design effect of 1.5 (see above), we will require a sample size of 272 mother-infant pairs in each of the intervention and comparison groups. Assuming a dropout rate of 25%, we need to recruit 680 mother-infant pairs (56-57 per subdistrict cluster).

#### Sampling Procedure

In the cross-sectional evaluation, the sampling design will follow a 3-stage cluster sampling procedure. In the first stage, in each of the 12 subdistricts selected for the study, we will randomly select 10 villages using the probability proportionate to size sampling method. In the second stage, we will select 2 villages from each village using simple random sampling. In the third stage, in each of the selected subvillages, we will list all households and conduct a mini census of children aged <2 years and pregnant women. Using this framework, we will use simple random sampling to select 12 children aged <2 years and their mothers and 2 pregnant women.

In the cohort evaluation, we will construct a sampling frame of all pregnant women in their third trimester from all the selected villages using the client lists of midwives and health volunteers. We will systematically contact the pregnant women on the list across all 6 subdistricts to obtain the required sample.

#### Recruitment and Consent

We have described the approach and criteria for selecting clusters in the section above on *Cluster Selection and Design of Randomization*. We will seek *Gatekeeper* consent [26] from the heads of the 2 study districts, the selected subdistricts, and the sampled villages.

In both the cross-sectional and cohort evaluations, we will recruit either women with children aged <2 years or pregnant women from the village-level sampling frames by contacting them at home. Trained field staff will explain in detail the background and objectives of the study to the women and provide a written information sheet to all women contacted. Written informed consent was obtained from women who agreed to participate in the study.

### Data Collection Methods

#### Data Collection Team

An organization not responsible for the intervention will recruit and manage a data collection team. For the cross-sectional evaluation, we will recruit 10 field coordinators, 130 interviewers, and 20 nurses or midwives to collect blood samples and to take anthropometric measurements. We will divide the staff into 10 field teams, with each team consisting of 1 field coordinator, 1 assistant field coordinator, 8 enumerators for interviews of mothers and caregivers of children aged <2 years, 2 interviewers for pregnant women, 1 interviewer for dietary data, and 2 anthropometry field workers who also collected the finger-prick blood sample for hemoglobin assessment using Hemocue. We will train the cross-sectional evaluation team for 1 week on an overview of the Baduta study, the use of the CommCare data capture app, household listing and data collection procedures, explanations of study instruments (listing forms and questionnaires), quality controls for data collection, and a field plan. The training will include a 1-day tryout to practice the household listing, interviews of mothers of children, and pregnant women using the CommCare data capture app.

For the cohort evaluation, we will recruit 2 field coordinators and 8 interviewers, all of whom will be graduates from a local nutrition academy. We will divide this staff into 2 field teams, 1 team for each district. We will train the cohort evaluation team for 1 week on an overview of the Baduta study, use of the CommCare data capture app, the schedule of cohort data collection, explanations of study instruments, anthropometry, dietary and hemoglobin assessment, and tryout of all the cohort data collection procedures. The 2-day training for blood collection will cover standard phlebotomy methods, use of Hemocue for hemoglobin assessment and its calibration, and a practice session in the field. The 2-day training for anthropometric measurements will be competency based and will use standardization procedures to check the performance (precision and accuracy) of the anthropometry field workers.

#### Data Collection Schedule

For the cross-sectional evaluation, we will conduct a household listing in each subcluster (hamlet) before the interviews to obtain a list of all children aged <2 years and pregnant women living in the subcluster. We will identify households by applying a sticker that has an identification number for each house listed. We will interview women at home but invite and accompany them and their selected children (aged <2 years) to a central location in the village to collect blood samples and record anthropometric measurements. We will use the same field methods for both the baseline and end-line surveys. We will conduct the end-line survey 2 years after the baseline survey.

For the cohort evaluation, the data collectors will visit each woman a total of 15 times from late in the third trimester of pregnancy until the child is 18 months old (Figure 3).

#### Types of Information Collected

For the cross-sectional evaluation, we will use the *house-listing form* (paper based) to record the identification number and collect household location and composition, including details about each member such as date of birth and gender. We will use a *registration module* (part of the CommCare app) to record the name and address of the potential respondents selected based on the house-listing form. This module will then direct interviewers to the appropriate questionnaire modules for children aged <2 years or pregnant women. The respondent will be either the mother and caregiver of the child aged <2 years or the pregnant women. The *questionnaires for children aged <2 years* (CommCare) will record the family sociodemographic characteristics, the history of pregnancy and delivery, the history of antenatal care services, exposure to the interventions (end line only), breastfeeding practices, use of integrated village health posts, morbidity, knowledge about breastfeeding, self-efficacy for breastfeeding, socioeconomic status, food security, and use of mobile phones.

Similarly, the *questionnaires for pregnant women* (CommCare) will record family sociodemographic characteristics, the history of pregnancy and delivery, exposure to interventions, knowledge about breastfeeding, knowledge about food to eat during pregnancy, intention to breastfeeding, socioeconomic status, and food security. We will use a special-purpose module (CommCare) to record the *anthropometric and hemoglobin measurements*. We will capture *dietary intake data* from children aged <2 years and pregnant women using the multiple-pass 24-HR (paper-based).

For the cohort evaluation, in pregnancy and until the child is 18 months old, the field staff will collect information about the location and composition of the household, pregnancy knowledge, breastfeeding intention, infant feeding knowledge, household food security, birth outcome and delivery services, antenatal care, IYCF practices, utilization of integrated village health post services and immunizations, exposure to Baduta interventions, child morbidity, maternal and child dietary intake, maternal and child anthropometry, and hemoglobin assessment (Figure 3). We will capture *dietary intake data* from the children and pregnant women using the multiple-pass 24-HR (paper based for the women and electronic data capture for the children).

#### Digital Data Collection Tool

We will capture data electronically on Android tablets in the field using a special-purpose CommCare app developed for the study. The interviewers will record the information on structured, error-detecting forms on tablets or mobile devices and dispatch it directly to a server for cleaning and merging. The app will navigate the interviewers through the different questionnaires for women with a child aged <2 years or pregnant women or the different visit forms in the cohort evaluation. We will generate and distribute routine field team performance reports based on the data captured to help supervisors manage field activities.

### Statistical Methods

The intention-to-treat principle will guide our data analysis, and we will include data from all participants even if they do not participate in all interventions. We will apply the guidelines of the Consolidated Standards of Reporting Trials statement for clustered randomized trials in the analysis and presentation of the results. We will conduct analyses at the woman or infant level adjusted for cluster randomization. Initially, we will assess the effectiveness of randomization by comparing potential confounding factors in both treatment groups. We will apply statistical tests with χ^2^ tests for categorical variables and an adjusted Wald test or the Fisher exact test for continuous variables to assess for any significant difference between treatment groups. *P* values <.05 will be considered to indicate a statistically significant difference between the groups. For selected dietary analyses of continuous variables, we will use the nonparametric rank order statistic, Somers’ D [51] (corresponding to rank-sum tests), as it can adjust for the cluster sampling design.

We will calculate frequency and percent distributions for categorical variables and mean and SD or median and interquartile range for continuous variables. We will characterize the dietary intake by age group, breastfeeding status (for infants aged 12-23 months), and intervention and comparison groups. We will generate medians and interquartile ranges for dietary intake distributions that are not normally distributed. We will plot the frequency distribution of IYCF practices by child age. We will also plot the frequency distribution of Z score indices (Z score curves) in 0.25 Z score intervals using a Lowes function to smooth the curves, and we will compare these distributions with the growth standard distribution. We will repeat these plots broken down by treatment groups.

For the cross-sectional evaluation, the primary analyses will compare the prevalence of stunting (low length-for-age<–2 Z score) and the 95% confidence intervals in each treatment group adjusted for clustering and any imbalanced baseline characteristics.

For the cohort evaluation, the models will include the treatment group as a fixed effect, the community cluster as a random effect to account for the cluster effect, and the impact of the interventions over time by testing for an interaction between time and intervention group. We will conduct analyses to identify the subgroups (eg, level of maternal education or household wealth categories) that modify the response to the intervention. We will check the model assumptions and make appropriate adjustments to the analysis where necessary. Secondary analyses will examine each outcome variable (length-for-age Z scores, feeding patterns, and mean nutrient intakes), taking into account the repeated measurements within children by using separate mixed models.

Linear mixed models will be used for continuous outcomes (eg, length-for-age Z scores), and generalized linear mixed models will be used for noncontinuous outcomes (eg, mixed logistic models for binary outcomes, such as, percentage exclusively breastfeeding). Models will include intervention or comparison groups as a fixed effect, infants as a random effect to account for repeated measurements, and community cluster as a random effect to account for cluster effects. We will use Stata V.15 (Stata Corp 2017; Stata Statistical Software: Release 15; Stata Corp LP) for all analyses.

### Research Ethics

We obtained ethical approval for the study from the Faculty of Public Health, University of Indonesia (323/H2.F10/PPM.00.02/2016) and the Human Research Ethics Committee of the University of Sydney (Protocol number: 2015/115). Trained field staff carefully explained the background and objectives of the study to the women and gave a written information sheet to all women contacted. We obtained written informed consent from the women who agreed to participate in the study. We will maintain the privacy, anonymity, and confidentiality of the information provided by respondents during all phases of the study. We will store all information in an encrypted database with the participant’s study identifier instead of personal identifiers, and only the investigators and authorized data management team will have access to the data collected.

### Data Monitoring

On the basis of the CommCare data capture system, we will design a real-time study monitoring information system to provide information on enrolment status, adherence of the data collectors to the evaluation schedule, and the quality of key data elements. The system will generate automated emails to distribute information to field supervisors. Field supervisors will use this system to monitor the performance of individual data collectors, and all reports will be available for review by the investigators.

### Data Access

All data collected will be accessible to the study investigators, who will have the right to analyze and publish data. We will make the relevant anonymized individual-level data available on reasonable request.

## Results

### Cross-Sectional Evaluation

We conducted the baseline survey for the cross-sectional evaluation in February 2015. We recruited 2435 mothers and their children aged <2 years, of whom 1199 were in the intervention group and 1236 were in the comparison group. Similarly, we recruited 409 pregnant women, of whom 198 were in the intervention group and 211 were in the comparison group.

We conducted the end-line survey in February 2017. We recruited 2740 mothers and their children aged <2 years, of whom 1357 were in the intervention group and 1383 were in the comparison group. Similarly, we recruited 642 pregnant women, of whom 306 were in the intervention group and 336 were in the comparison group.

### Cohort Evaluation

We began recruitment to the cohort evaluation in February 2015 and completed the follow-up of participants in December 2016. We screened 729 pregnant women for their eligibility to join the study, of which 685 mothers (343 in the control group and 342 in the intervention group) who delivered their babies were followed up in the study till the child was 18 months old. Among them, 331 in the control group and 327 in the intervention group remained in the study until the end. There were 12 dropouts in the control group and 15 dropouts in the intervention group.

We completed data cleaning and processing for both evaluations in July 2017, followed by analysis, report writing, and dissemination of the results.

## Discussion

### Overview

Childhood undernutrition remains a global public health nutrition problem, with an estimated 150 million children worldwide with stunted linear growth, despite recent progress made to improve the nutritional status of children [52]. Although Indonesia is a middle-income country, it is ranked 25th globally for its prevalence of childhood stunting and fifth for the number of stunted children [53]. Furthermore, the prevalence of stunting among children aged <5 years in Indonesia between 2007 and 2013 has remained stagnant at 36%-37% [54]. The Government of Indonesia has established nutrition development targets and aims to reduce the prevalence of stunting among children aged <2 years from 33% in 2013 to 28% in 2019 [22]. Strong evidence is needed about effective intervention packages to reduce stunting to guide policy and program decisions at the national level and for the decentralized district governments. This paper describes a study protocol of a cluster randomized controlled study to assess the impact of a package of behavior change interventions and health system strengthening activities on maternal and child nutrition status.

### Limitations

We designed the study to examine the impact of the package of Baduta nutritional and behavioral change interventions on the primary outcome, child stunting; therefore, we cannot identify the impact of the individual behavior change intervention components. We measured the exposure to the interventions in the end-line survey for the cross-sectional evaluation and at the end of the follow-up period at 18 months in the cohort evaluation. This information is for process evaluation, but we may use it to examine the associations between the individual intervention components and child growth in analytical epidemiological analyses.

The small number of clusters in which it was feasible to implement the interventions, especially the health systems interventions, prevented the use of simple randomization to allocate the treatments. We addressed this study design limitation through the use of restricted randomization [26,30] in which we used existing village census and health surveillance information to identify the set of combinations of the study subdistrict clusters that had a balance of characteristics or indicators that were related to the study outcomes. We randomly selected one of these balanced combinations to allocate study treatments.

A further limitation of the study is the lack of an economic analysis to assess the cost-benefit of the program for preventing child stunting.

### Study Implications

In Indonesia, reaching the current nutrition targets for child stunting will require an integrated approach that includes support for appropriate IYCF practices, including exclusive breastfeeding for 6 months, continued breastfeeding for 2 years, timely and appropriate complementary feeding, improved maternal nutrition, access to quality health services, water and sanitation, and other public health measures. The results of this study will provide valuable inputs for future research on preventing child stunting and help to formulate and guide policies to support effective packages of nutrition behavior change interventions to prevent child stunting in Indonesia.

## Appendix

Multimedia Appendix 1 Map of Baduta impact evaluation districts in East Java.

Multimedia Appendix 2 Description of emo-demo sessions.

Multimedia Appendix 3 Emo-demo visuals.

Multimedia Appendix 4 Description of the four TV spots.

Multimedia Appendix 5 Baduta behaviour change messages in poster format.

## Abbreviations

24-HR: 24-hour dietary recalls
DHS: Demographic and Health Survey
GAIN: Global Alliance for Improved Nutrition
Hb: hemoglobin
IYCF: infant and young child feeding
SEAMEO-RECFON: Regional Centre for Food and Nutrition, Southeast Asian Ministers of Education Organization
UNICEF: United Nations Children’s Emergency Fund
WHO: World Health Organization
