# Supplementary figures and images for: Evaluation of a Package of Behaviour Change Interventions (Baduta Program) to Improve Maternal and Child Nutrition in East Java, Indonesia: Protocol for an Impact Study
Source: JMIR Res Protoc. 2020 Sep 8;9(9):e18521. doi: 10.2196/18521 (PMC7509610; doi:10.2196/18521)

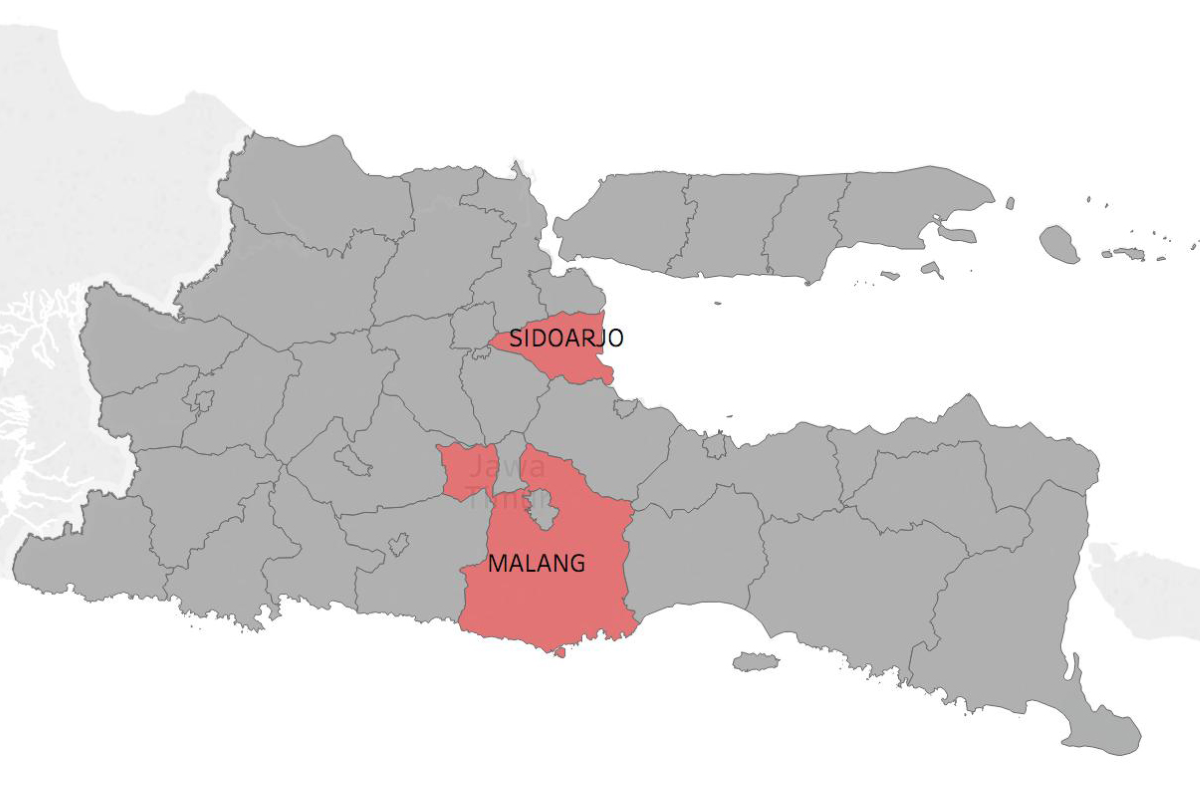

Supplement: Multimedia Appendix 1 [file resprot_v9i9e18521_app1.png]

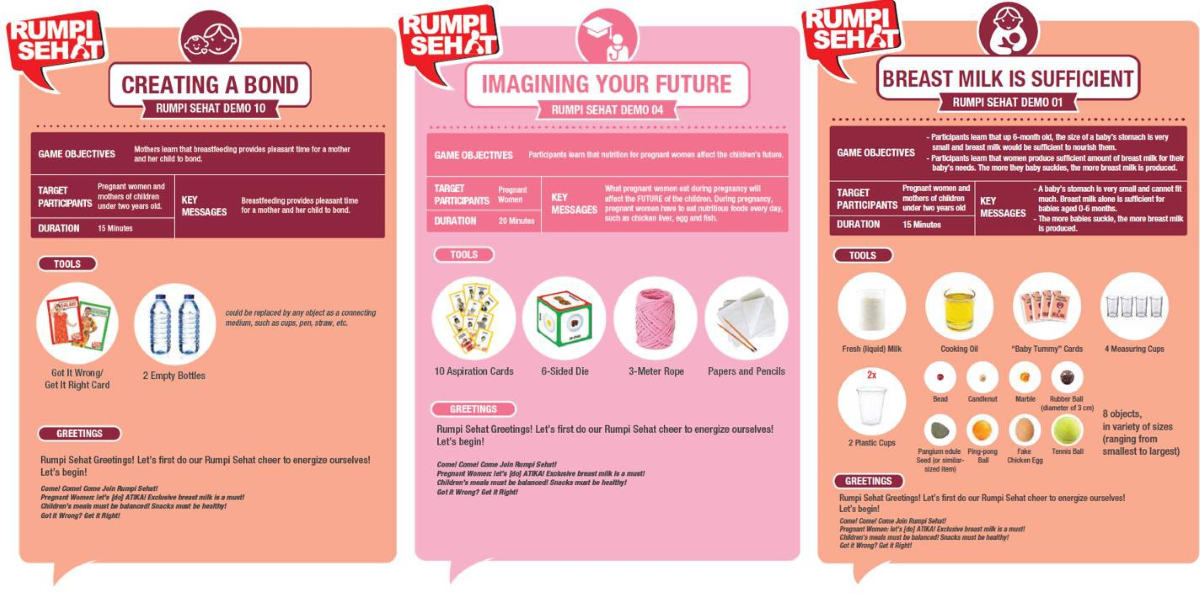

Supplement: Multimedia Appendix 3 [file resprot_v9i9e18521_app3.png]

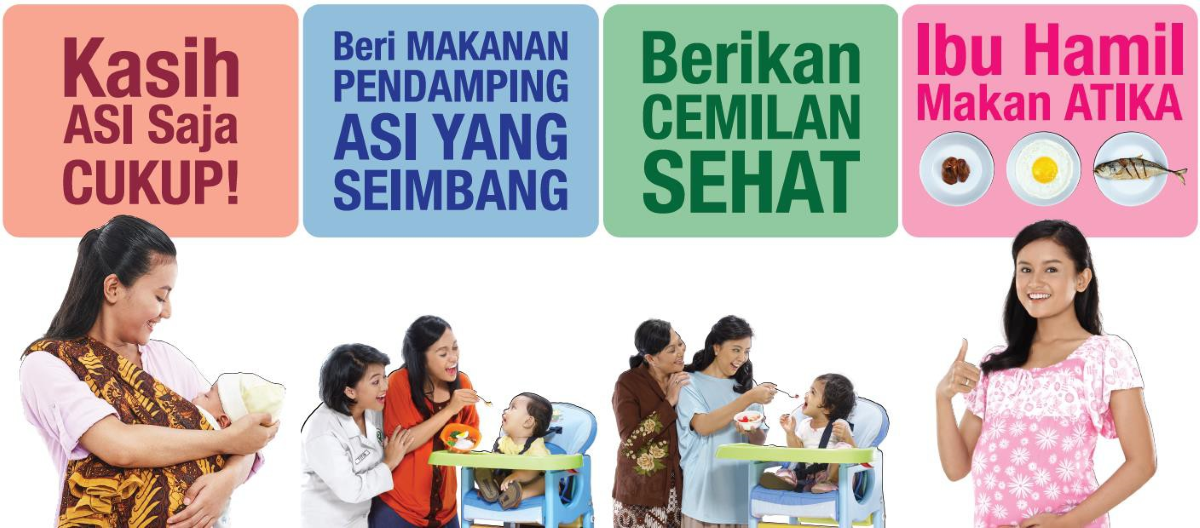

Supplement: Multimedia Appendix 5 [file resprot_v9i9e18521_app5.png]
