# Supplementary material for: Evaluation of a Package of Behaviour Change Interventions (Baduta Program) to Improve Maternal and Child Nutrition in East Java, Indonesia: Protocol for an Impact Study
Source: JMIR Res Protoc. 2020 Sep 8;9(9):e18521. doi: 10.2196/18521 (PMC7509610; doi:10.2196/18521)
Supplement: Multimedia Appendix 2 [file resprot_v9i9e18521_app2.docx]

Multimedia Appendix 2: Description of Emo-Demo sessions

| Emo-Demo Session | Description |
| --- | --- |
| Sessions for pregnant women: | |
| 1. Ati, Telur, Ikan – “Atika” (Liver, Eggs, Fish) (“Secrets of strengthening foods”) | Pregnant women measure and compare the amount of rice and Krupuk (a popular fried cracker) that they would need to consume to obtain the same amount of iron required during pregnancy as consuming an egg, fish, or liver. This session dramatizes the benefits of animal source foods, specifically liver, eggs, and fish, and encourages a pregnant woman to eat a serving every day. |
| 1. Ati, Telur, Ikan – “Atika” (Liver, Eggs, Fish) (“Imagining your future”) | Pregnant women choose a card picturing small children dressed in aspirational professional uniforms (such as a doctor or police officer) and roll a dice with different foods on it. Pregnant women are paired and stand apart 2-3 meters (depending on available space) connected with a plastic rope they hold. One mother also holds the card, and one mother on the other side throws a big dice with three pictures of the liver, eggs, and fish, and on the other side pictures of unhealthy food. They get to move closer to their picture card, depending on the food they land on. They can take the most steps forward when they land on animal source foods such as liver, eggs, and fish. The session emphasizes the relationship between a pregnant woman’s diet that incorporates animal source foods, and infant growth and development, and attempts to trigger an emotional driver. by imagining their child’s future |
| 1. Healthy eating during pregnancy (“Seeing you for the first time”) | Participants receive a drawing of a fetus’s development at the gestational age of their pregnancy with a brief description of the fetus’s state of development. Participants then write a message with wishes for their unborn baby. The group talks about how they can support their baby’s optimal growth and development through eating right. Writing the message and seeing the state of development raises the emotional stakes. |
| 1. Emotional bonding through breastfeeding (“Creating bonds”) | The facilitator arranges the pregnant women in pairs. However, instead of shaking hands, they touch each other’s hands using a material, e.g., water bottle or pencil, and share a story without physically touching each other. Then the pairs tell each other a story while holding hands and purposefully making eye contact. The session illustrates the increased closeness between two people when there is skin/eye contact, and they compare this experience to breastfeeding. |
| 1. The convenience of breastfeeding (“Getting ready to go out”) | Pregnant women participants volunteer to role-play a breastfeeding mother and a mother who is formula feeding. The women must race each other to prepare all the items they need to go out of the house with their babies. One volunteer counts the time to show who is quickest in getting ready. The session is a fun way to demonstrate that breastfeeding is more convenient than formula feeding because there is no need to prepare formula and bottles. |
| 1. Care for pregnant women (“Being pulled in all directions”) | A volunteer represents an assigned fictional pregnant woman. Other participants read cards with problems that she faces during pregnancy (e.g., not feeling well physically, having older children to look after, her husband working in another city, work problems.). A string attaches each card to the volunteer in the center of a circle. Other participants pull strings to illustrate how pregnant women are “pulled in all directions” and do not get enough care and support. The session communicates the stress that many women feel during pregnancy and emphasizes the need to look after oneself, including eating well and seeking health care when one does not feel well. |
| Sessions for caregivers of children 0-23 months of age: | |
| 1. Breastmilk is sufficient (“Baby Tummy”) | Pregnant women guess the size of a baby’s stomach at different ages beginning at one day, three days, one week, and one month, using different sized round objects (stone, nut, marble, egg). The session aims to overcome the fear that breastmilk is not sufficient to satisfy baby’s needs by demonstrating the small size of a baby’s stomach and explaining why a baby may eat a small amount and also why it needs frequent feeds. The facilitator shows two glasses, one representing the baby and the other mothers’ breasts, to demonstrate that as much as the baby drinks, the breasts produce a similar amount of milk to replace it. The facilitator does the same in the other two glasses but reduces the amount of milk then adds some oil into the baby’s glass to represent formula. The oil naturally forms a separate, visible layer. The facilitator then explains that the mother who only breastfeeds will produce more breastmilk, while the mother who also gives formula will not replace that amount, so there will be less milk produced than the baby has drunk. Adding more formula reduces breast milk production. The session aims to demonstrate how breastmilk production increases to meet the baby’s needs and to discourage formula use. |
| 1. Complementary foods (“Ideal plate) | The facilitator asks the participants to show the portions of rice, vegetables, and fruits, and a side dish or other protein resource foods fed their children were during the previous day using color-coded wedges on a plate. The facilitator then demonstrates the “ideal plate,” including the proportion of rice and other foods to feed to a child. This highly interactive session promotes reducing the portion of rice and increasing the portions of vegetables, fruits, or protein (animal/plants) source foods fed to infants and young children. |
| 1. Healthy snacking (“Unhealthy /unsafe snacks”) | Unhealthy snacks – to simulate the snacks consumed and their appearance in the stomach of a child, the facilitator mixes mostly puff snacks (in Indonesia, typically containing many artificial chemicals and coloring) with hot water and leaves in a transparent cup for a while. While the snacks dissolve in the water, two volunteers compete to settle a crying baby (represented by a bucket) by throwing snacks (balls) into it every time the facilitator indicates that the baby begins to cry. In the end, there is a surprise result with the winner being the woman who has the least (and not the most) balls in the bucket. The session discusses avoiding giving children unhealthy snacks every time they cry. Then, the participants pass around the cup and consider its appearance, and if they would want to feed the contents to their babies. This session aims to elicit a feeling of disgust for unhealthy snacks and promote feeding healthy snacks to children. |
| 1. Timing of meals (“Feeding timeline”) | Participants create and discuss a daily timeline for feeding their children including breastfeeding, meals, and snacks by putting cards picturing a breastfeeding mother, a plate of food (meal), and a plate of fruit (as a snack) on the timeline banner. This session promotes breastfeeding on demand and not giving snacks to children immediately before meals. |
| 1. Relationship between feeding habits and growth (“Building Blocks) | Mothers race to build the tallest “child” from blocks by reading cards with points about child feeding practices. The cards contained points for positive feeding practice and 0 for harmful practices. This session promotes the importance of feeding children a healthy, balanced diet for optimal growth. |
| 1. Handwashing with Soap | The facilitator demonstrates how germs transfer from one material to another using glow powder as fake germs and a UV lamp> this demonstration includes the participant’s hands even if they appear clean. This session promotes the importance of handwashing with soap, three important moments to wash hands, and steps on handwashing with soap. |

This document is a Multimedia Appendix to a full manuscript published in JMIR Research Protocols For full copyright and citation information see http://dx.doi.org/10.2196/jmir.xxxx
