# Supplementary material for: Evaluation of a Package of Behaviour Change Interventions (Baduta Program) to Improve Maternal and Child Nutrition in East Java, Indonesia: Protocol for an Impact Study
Source: JMIR Res Protoc. 2020 Sep 8;9(9):e18521. doi: 10.2196/18521 (PMC7509610; doi:10.2196/18521)
Supplement: Multimedia Appendix 4 [file resprot_v9i9e18521_app4.docx]

Multimedia Appendix 4: Description of TV Spots

| TV Spot | Description |
| --- | --- |
| 1. Nutrition during pregnancy-focused: *Ati, Telur, Ikan – “Atika.”* | A man preparing for work tells a gossipy neighbor that the vegetables his wife has packed for his lunch are enough for him but that his pregnant wife needs different food. The gossipy neighbor says she only ate fruits and vegetables while pregnant. The mother-in-law comes back from the market with chicken liver for her pregnant daughter-in-law and tells the gossipy neighbor that during pregnancy, especially the first three months, a woman needs to eat at least one portion of chicken liver, egg, or fish a day to remain strong and energetic during pregnancy. This spot promotes incorporating animal-source foods, specifically liver, eggs, and fish, into a pregnant woman's diet. |
| 1. Exclusive breastfeeding | A father and shopkeeper are complaining about the hassle and cost of preparing formula milk. His friend says that his wife is exclusively breastfeeding, which is sufficient for the baby. The wife, mother-in-law, and baby arrive, and the mother-in-law throws the baby bottle in the rubbish bin, saying, "If you bottle feed, you produce less breastmilk. The more you breastfeed your baby, the more you produce breastmilk." This spot emphasizes that mothers produce enough breastmilk and that breastmilk is all babies need for the first six months of life. |
| 1. Complementary foods | A mother is buying fruit and vegetables for her baby from a street vendor. The gossipy neighbor says that another mother gives her child rice-meals, which should not be enough. The health worker says the mother is right, and a balanced meal is very important, a child is different from an adult. This spot promotes given a diversity of foods to ensure optimal growth and development |
| 1. Healthy snacking | A gossipy neighbor tries to give an unhealthy fried snack to a baby toddler. The nearby health worker says the crisps have no nutrients and will spoil the baby’s appetite. The baby’s mother comes with a bowl of fruit and shares that one should not give a snack just before a meal, and the snack itself should be healthy. This spot promotes providing healthy snacks and not junk food to children. |

This is a Multimedia Appendix to a full manuscript published in JMIR Research Protocols For full copyright and citation information see http://dx.doi.org/10.2196/jmir.xxxx
